# An anonymization-based privacy-preserving data collection protocol for digital health data

**DOI:** 10.3389/fpubh.2023.1125011

**Published:** 2023-03-03

**Authors:** J. Andrew, R. Jennifer Eunice, J. Karthikeyan

**Affiliations:** ^1^Computer Science and Engineering, Manipal Institute of Technology, Manipal Academy of Higher Education, Manipal, Karnataka, India; ^2^Electronics and Communication Engineering, Karunya Institute of Technology and Sciences, Coimbatore, Tamil Nadu, India; ^3^School of Information Technology and Engineering, Vellore Institute of Technology, Vellore, Tamil Nadu, India

**Keywords:** anonymization, data privacy, healthcare data, *k*-anonymity, privacy-preserving, data collection

## Abstract

Digital health data collection is vital for healthcare and medical research. But it contains sensitive information about patients, which makes it challenging. To collect health data without privacy breaches, it must be secured between the data owner and the collector. Existing data collection research studies have too stringent assumptions such as using a third-party anonymizer or a private channel amid the data owner and the collector. These studies are more susceptible to privacy attacks due to third-party involvement, which makes them less applicable for privacy-preserving healthcare data collection. This article proposes a novel privacy-preserving data collection protocol that anonymizes healthcare data without using a third-party anonymizer or a private channel for data transmission. A clustering-based *k*-anonymity model was adopted to efficiently prevent identity disclosure attacks, and the communication between the data owner and the collector is restricted to some elected representatives of each equivalent group of data owners. We also identified a privacy attack, known as “leader collusion”, in which the elected representatives may collaborate to violate an individual's privacy. We propose solutions for such collisions and sensitive attribute protection. A greedy heuristic method is devised to efficiently handle the data owners who join or depart the anonymization process dynamically. Furthermore, we present the potential privacy attacks on the proposed protocol and theoretical analysis. Extensive experiments are conducted in real-world datasets, and the results suggest that our solution outperforms the state-of-the-art techniques in terms of privacy protection and computational complexity.

## 1. Introduction

Healthcare industries have seen a significant transition since the advancements in communication technologies (1). E-health services (2) have become popular for their wide range of advantages such as accuracy, timeliness, easy access, and efficiency (3, 4). Electronic health records (EHRs) (5) are the major step toward the transformation of traditional healthcare services into paperless medical practice that can reduce the risk of medical errors (6–8). Digitized patients' health record benefits both patients and healthcare providers in sharing, monitoring, tracking, and analyzing the healthcare of patients (9). As EHRs follow a standard health record format, it is possible to make them available worldwide (10). EHRs reduce administrative overhead, costs, and medical errors through efficient communication of health information (11). Healthcare organizations often collect EHRs for medical and research purposes (12). EHRs generally contain information concerning individual health records, medical history, medications, physical conditions, etc. (13). Since there is a huge amount of personal information contained in EHRs, it is crucial to consider privacy issues more carefully (14–16).

Collecting personal health records without breaching the privacy of involved individuals is essential for its success (17–20). In the data collection problem, the data collector is usually an untrusted third-party service provider who collects data from a set of individual data owners (21, 22). Assume that a medical researcher requests data from a number of patients who hold the healthcare demographics. The schema of demography consists of user ID, age, sex, weight, and diagnosis that every patient provides to the data collector. The health record schema is a combination of personal identifiers (e.g., user ID), quasi-identifiers (QI) (e.g., age, sex, weight), and a sensitive attribute (e.g., diagnosis). A sample healthcare records collection table is shown in Table 1.

**Table 1:** Electronic health records.

**User ID**	**Age**	**Sex**	**Weight**	**Diagnosis**
1,2,3	30–40	F	55	Gastritis
		F	50	Flu
		F	60	Dyspepsia
4,5,6	55–65	M	65	Pneumonia
		M	75	Flu
		M	68	Cancer

In the aforementioned example, although there are no direct identifiers such as name and social security number (SSN) in the EHR, privacy breaches can still arise. An untrusted data collector can ascertain the identity of the patient through the explicit identifier *userID* and sensitive attribute *diagnosis* of each individual. Although QI cannot be used to directly identify a person, by connecting them to the data in a published database, it may be possible to do so. The QI can act as an identifier in the absence of a direct identifier. Hence, identity disclosure is one of the major privacy issues in EHR. In the data collection problem, identity disclosure (23) can arise both at internal and external levels. Internal identity disclosure (24) generally happens within the organization either through the data owners or the data collectors. External identity disclosure (25) takes place when the data is transmitted between the owner and the collector.

Unsurprisingly, privacy-preserving healthcare data collection has become a recent research focus where a good number of literature exists (26–32). Cryptography or anonymization-based approaches are widely used to prevent the identity disclosure of EHR (33, 34). Symmetric key and asymmetric key cryptography, multiparty computation, and homomorphic encryption are some of the cryptographic approaches used for privacy-preserving data collection (35); although it guarantees privacy to a certain extent, significant challenges such as heavy computation and key propagation make it a difficult choice. The anonymization approach (36), in general, removes the identifiers and generalizes the QIs excluding the sensitive attribute. Traditional anonymization techniques, such as *k*-anonymity (37), *l*-diversity (38), *t*-closeness (39), clustering-based *k*-anonymity (40), (α, k)-anonymity (41), *p*-sensitive *k*-anonymity (42), and others, anonymize the personal records by grouping similar QI attributes to make them indistinguishable from other sets of records in the same table.

Most of the literature for privacy-preserving data collection has not considered distributed data owners, and it is assumed that personal data are already collected in a common place to be anonymized (43). Hence, in centralized solutions for privacy-preserving data collection, it has become essential to employ a third-party anonymizer (44). However, it is highly undesirable for a patient to share his/her original EHR with a third party. There is also a huge risk of a privacy breach when a data owner (patient) directly shares their personal information with the data collector. The existing privacy models drudged to control the disclosure by deploying an anonymization layer or private unidentified channel between the data collector and the data owner. Nonetheless, such assumptions are not practical as the layer or channel is not persistent. Cryptographic approaches also encrypt the healthcare records to prevent identity disclosure at the data collector's end; furthermore, the data are anonymized, resulting in poor data utility.

In this research, we propose a data collection protocol for EHRs that is effective and protects privacy in order to address the aforementioned problems. In the proposed protocol, multiple data owners anonymize their health records in a distributed and collaborative fashion before submitting the data to the data collector. This protocol's main goal is to forbid explicit exchanges between data owners and data collectors. The data owners submit their anonymized QIs through a set of representatives elected for their equivalent group. Representatives are data owners of the equivalent group with common quasi attributes. Every equivalent group should satisfy the clustering-based *k*-anonymity property (i.e., at least *k*-1 records share the same quasi attributes); therefore, the anonymized records with common QIs are submitted to the data collector through group representatives. This approach of the proposed protocol is efficient in tackling internal and external identity disclosure. Table 1 shows the original EHR of *n* patients, Table 2 shows the anonymized version of the original records by the proposed protocol. As shown in Table 2, there are two equivalent groups that share common QIs of size *k* = 3. Such equivalent groups, along with sensitive values (e.g., diagnosis), are collected by the data collector, which reduces the risk of identity disclosure. Furthermore, dynamic data owners who join or leave an equivalent group are handled by a greedy heuristic method.

**Table 2:** 3-anonymized health records.

**User ID**	**Age**	**Sex**	**Weight**	**Diagnosis**
1	35	F	55	Gastritis
2	40	F	50	Flu
3	45	F	60	Dyspepsia
4	55	M	65	Pneumonia
5	60	M	75	Flu
6	65	M	68	Cancer

The major contributions of the proposed protocol are as follows:

(1) **Privacy-preserving healthcare data collection protocol:** A novel *k*-anonymity-based data collection protocol specifically for healthcare data collection is proposed.
(2) **Leader election:** A leader election algorithm is proposed to elect representatives of equivalent groups of anonymized records that share similar generalized quasi attributes.
(3) **Greedy heuristic method:** Data owners who dynamically join or leave the group is efficiently managed without affecting the data utility and privacy.
(4) **Leader collision mitigation and sensitive attribute protection:** We propose solutions for privacy breach through leader collision and methods to enhance the protection of sensitive attributes.

The remainder of this article is structured as follows. The recent state-of-the-art literature is discussed in the Section 2. In the Section 3, an adversarial model of the proposed protocol is presented, along with a data model and other definitions. In the Section 4, the proposed protocol is formally defined, along with the proposed algorithms. In the Section 5, data utility and possible privacy attacks on the proposed protocol are discussed. In the Section 6, experiments conducted are presented. Finally, the Section 7 concludes the article.

## 2. Literature survey

In the last decade, a huge number of research studies were conducted in privacy-preserving data publication and data collection. This section presents a detailed study of various state-of-the-art literature available in the field of preserving the privacy of personal data. In privacy-preserving data collection and publication, disclosure or reidentification of data owners has been a significant issue. The state-of-the-art literature consists of cryptographic and anonymization-based approaches for privacy preservation. The collection of personal data is accomplished through devices and sensors. The device periodically collects and transmits the data to the data collector upon request. The data transmission is generally conducted in a closed or open network. Hence, it is essential to ensure the secure transmission of data. Hussien et al. (45) used a symmetric key cryptographic technique to propose a secure and energy-efficient method to collect data in wireless sensor networks.

Most privacy-preserving schemes require a secure transmission channel or a third-party authentication system. However, they are impractical due to various challenges. In (46), Beg et al. have proposed a reversible data transform (RDT) algorithm for privacy-preserving data collection in the mobile recommendation system (MRS). The proposed RDT algorithm is used to protect sensitive attributes. To avoid the third-party role in the data collection process, the data transfer is done through elected representatives. However, the leader election process is straightforward, and leader collision is possible that can breach privacy. However, the same authors in (47) addressed the RDT prior data sharing and its parameter protection challenges by proposing a chaotic RDT for PPDP MRS. The authors also claim that the proposed approach can replace homomorphic encryption techniques and preserve the privacy of the MRS. The leader collusion problem is addressed by Sajjad et al. (48) through a random leader election mechanism that elects the leaders randomly and maintains a leader table for maintaining the records. However, this scheme is inefficient, which simply uses a random function to select the leaders, and leader collusion is still possible when the number of available groups is minimal. Data anonymization is vital in protecting big data and IoT data. Ni et al. (49) evaluated the performance of data anonymization schemes in an IoT environment for big data. The authors addressed the reidentification risks and evaluated the schemes based on privacy preserving-level and data utility metrics. Traditional anonymization schemes like *k*-anonymity, *l*-diversity, obfuscation, permutation, and differential privacy techniques (50) are evaluated through information loss, data utility, and conditional entropy. A similar study was presented by Sun et al. (51) for trajectory data publishing. Canbay et al. (52) proposed a Mondrian-based utility aware anonymization approach called u-Mondrian. This approach is aimed to address the upper-bound problem in the Mondrian anonymization approach that leads to poor data utility.

Healthcare data contain sensitive information that must be protected concurrently; it is very vital for healthcare research. Hence, it is essential for protecting the privacy of healthcare data with appropriate data utility. In (53), we proposed a clustering-based anonymization approach for privacy-preserving data collection in a healthcare IoT environment. The proposed approach utilizes a client–server model to anonymize the healthcare data before it reaches the data collector. The model is evaluated with information loss and other data utility metrics. A similar approach was proposed by Abbasi and Mohammadi (54) to protect the privacy of healthcare data in cloud-based systems. They proposed an optimal *k*-anonymity technique called the *k*-means++ method and used the normal distribution function to improve the anonymization data utility. We performed another study called an attribute-focused approach (55) to protect the privacy of healthcare data during data publishing. In this study, the healthcare attributes are categorized as numerical and sensitive attributes. A fixed-length interval approach is used to protect the numerical attributes and an improved *l*-diversity approach is used to protect the sensitive attributes. Avraam et al. (56) proposed a deterministic approach for protecting the privacy of sensitive attributes. This approach identifies the categorical and continuous attributes from the dataset and applies different mechanisms to prevent a privacy breach. The stratification technique is used for categorical and continuous attributes that are redistributed based on *k*-nearest-neighbor algorithms. The proposed approach is claimed to be efficient in preventing the data from reidentification. Kanwal et al. proposed multiple anonymization-based approaches to preserve the privacy of health records. In (57), they proposed a privacy scheme called horizontal sliced permuted permutation to protect multiple records of data owners. They considered the protection of multiple sensitive attributes by proposing 1: M MSA-(p, l)-diversity approach (58). Furthermore, the authors proposed an anonymization technique with an access control mechanism for hybrid healthcare cloud services. In all the studies, they evaluated data privacy for various privacy attacks such as identity disclosure attacks, membership disclosure, and sensitive attribute disclosures. Jayapradha and Prakash (59) presented a privacy-preserving model called *f*-slip that uses a frequency-slicing approach to protect sensitive attributes. Sensitive attributes are correlated to maintain the linking relationship during the anonymization process. Khan et al. (60) used phonetic encoding and generalization approaches for record linkage problems. The authors used phonetic encoding for anonymizing textual data, and for categorical and numerical attributes, the *k*-anonymization-based approach is utilized. Raju and Naresh (61) proposed a distributed algorithm to merge the datasets from different sources to maintain their privacy. To preserve the privacy of the sensitive attributes, they proposed a bucketization-based approach called (l,m,d)^*^- anonymity. The proposed approach anonymizes the data and transforms the data into a sensitive attribute and quasi-attribute table.

Based on the in-depth literature study of the recently published literature, most of the privacy-preserving models are still using the *k*-anonymization-based approach. However, they either use a private secure channel or a third-party anonymizer for privacy-preserving data collection. This may lead to a possible privacy breach. Hence, a *k*-anonymity-based privacy-preserving protocol for data collection without a third-party anonymizer is on demand.

## 3. Preliminaries

Various terminologies used in this study are introduced in this section. The components of the proposed protocol such as the data model, adversary model, and system architecture are defined.

### 3.1. Data model

We assume that EHRs are generated periodically on the users' devices. Out of the different attributes of personal healthcare data, only the major attributes such as personal identifiers, QIs, and sensitive attributes are considered in this article. Personal identifiers are explicit attributes that unambiguously distinguish a particular individual (e.g., social security number, name, IP address, and phone number). Identifiers are generally removed in the process of data collection and publication to avoid identity and attribute disclosure.

QIs are common attributes that can be shared by more than one data owners (e.g., age, sex, and zip code). Although they cannot directly identify an individual, the combination of QIs with publicly available datasets may breach privacy. In general, generalization and suppression approaches are used to protect QIs. Sensitive attributes (S) are details about a person that should not be shared (e.g., diagnosis). Identification of an individual's sensitive information, along with the identity, is a serious privacy breach. Hence, sensitive information is needed and protected with top priority.

#### 3.1.1. Definition 1: (Personal health data)

In personal health records table *T*, let *H* be a unique record in the table and *H*^*qi*^ be one of the QIs, and *H*^*si*^ be the single sensitive attribute (S) of the particular record. The health data schema is then defined as follows:


$$\begin{array}{l} \left(H_{1}^{qi},H_{2}^{qi},H_{3}^{qi},\ldots ,H_{m}^{qi},H^{si}\right) \end{array}$$

where *m* is the number of QIs for the record. In this article, a single sensitive attribute problem is considered.

#### 3.1.2. Definition 2: (Anonymization)

The term anonymization means protecting the identity. Hence, it involves a process of transforming the original health records to an equivalent less significant record. The original health record table *T* is mapped with an anonymization function *f* to generate an anonymized table T^*^. Every record of *t* in *T* is mapped to a record in T^*^. The anonymized QI attribute QI^*^ for every *t* in T^*^ is then defined as $t_{i}\left[QI\right]≺t_{i}^{*}\left[QI\right]$.

#### 3.1.3. Definition 3: (*k*-anonymity)

A personal health dataset *T* satisfies *k*-anonymity when a record *t* of T^*^ is imperceptible from at least *k*-1 other records. It is given by *k* ≤ *N*(*t*(*QI*)) for every record *t* ∈ *T*, *N*(*t*(*QI*)) – number of records shares the same QI.

#### 3.1.4. Definition 4: (Clustering-based *k*-anonymity)

A personal health dataset *T* satisfies the clustering-based *k*-anonymity (25) property if a set of clusters formed from *n* records where each cluster consists of *k* records where *k* ≤ *n*.

#### 3.1.5. Definition 5: (Equivalence class)

To create an equivalent class, at least k data owners' anonymized records with related quasi characteristics must be used. Let *G*^*E*^ represent the collection of data owners *k* who are grouped by the same anonymized quasi attributes QI^*^. *G*^*E*^ is an equivalent group if and only if *G*^*E*^ = {*d*|*d*[*QI*] = *qi*} and *k* ≤ *G*^*E*^, where *d* represents an arbitrary data owner with quasi attribute *d*[*QI* ].

### 3.2. Adversary model

In privacy-preserving healthcare data collection context, there could be a single data collector and multiple data owners.

Personal health data are generated by data owners (Definition 1). We assume that there are *n* data owners in the network and can communicate with other data owners and the collector. The client devices (e.g., medical sensors) at the data owner's end perform communication. The data owners collaborate with other clients not only to protect their health data but also patients in the network.

The data collector collects anonymized health records from the patients. In our protocol, the data collector is assumed to be a single semi-honest collector in the network. A semi-honest entity in a network generally follows the protocols but sometimes breaches the protocol to acquire more information. An attempt may be made to learn more about a person by a semi-honest data collector. This leads to identity disclosure.

A group of data owners who share the same quasi attributes forms an equivalent group (Definition 5) satisfying the *k*-anonymity and clustering-based *k*-anonymity model (i.e., at least *k* data owners in an equivalent group). Table 2 shows the example of an anonymity model that contains two groups with the value of *k* = 3. The records in the equivalent group share similar quasi attributes. The data owners interact with the data collector through the equivalent groups. Thus, it protects the data from external identity disclosure. Since the data owners share common quasi attributes in an equivalent group, internal identity disclosure is also protected.

An adversarial model is necessary to identify possible privacy attacks in the system. In a privacy-preserving data collection model, an adversary could be a data collector and data owner. The data collector is considered to be a malicious component in the network. Therefore, giving the data collector access to the original records is not appropriate. The clustering-based *k*-anonymity model ensures anonymized data is submitted to the data collector. The data owner can also be an adversary. An adversarial data owner generates fake quasi attributes and gets added to a specific equivalent group. During the random election of group representatives, if the adversarial data owners are elected as the first and second leaders of the group, then the sensitive attributes are disclosed. Such an attack is called a leader collision attack (LCA).

### 3.3. Overview of the protocol

Initialization, leader election, and data collecting phases make up the proposed data collection process. In the initialization step, the data owners (patients) create QI attributes and provide them to the data collector (without sensitive attributes). The data collector applies the provided clustering-based k-anonymity model to anonymize the health records. This results in the original QI being equivalent to at least *k*-1 generalized quasi characteristics (GQI). The appropriate data owners are then given the GQI and the list of data owners. The data owners then create comparable groupings that comply with the privacy policy.

**Figure 1:**
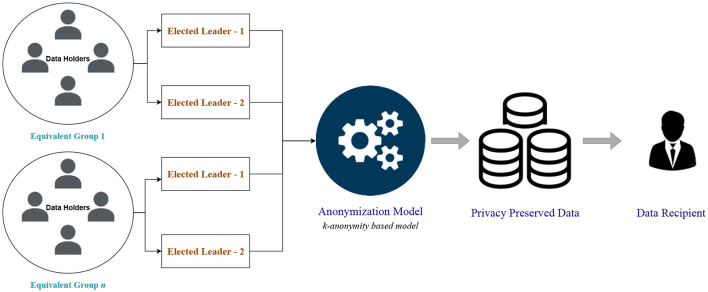
Privacy-preserving data collection protocol architecture.

In the leader election phase, members of an equivalent group are assigned with unique numbers; then based on a random number generation function, two leaders are elected for each equivalent class. The first leader obtains each member's hidden sensitive attributes from the phase of data collecting that uses sensitive values that are not real. The GQI and list of sensitive data are then given to the data collector. Without actually possessing sensitive information, the second leader gathers counterfeit sensitive information. In order to obtain the anonymized dataset, the data collector then executes intersection operations on the first and second leader datasets. The proposed privacy-preserving data collection protocol's architecture is depicted in Figure 1.

The proposed approach additionally takes into consideration of dynamic data owners who join or depart the equivalent class during the anonymization process. Dynamic join or leave follows the privacy requirement and ensures the required number of members for each group.

## 4. Privacy-preserving healthcare data collection protocol

Initialization, leader election, and data collection are the three phases of the protocol. The anonymization network is organized during the initiation phase, and the QI properties of the data owners are generalized. Representatives from related groups were chosen to serve as the leader during the election process. The data collector is finally given access to the anonymized records with quasi characteristics and sensitive attributes during the data collecting phase. We also outline techniques for managing data owners who join or leave the network on a dynamic basis.

### 4.1. Initialization phase

The anonymization network is set up by the initialization phase. Data owners and data collectors are required to initialize their attributes for the network. There are two algorithms proposed for data owner initialization and data collector initialization. Data owners initially transmit their QI attributes to the data collector over the specified network. It should be highlighted that the data owners do not send their sensitive qualities. Over time, the data collector gets QI attributes from *n* data owners. Then the data collector anonymizes the QI attributes based on any given privacy model (37–40) to generate generalized quasi attributes (GQI). For example, Table 1 shows the original health records of *n* (*n* = 6) data owners that are sent to the data collector without the sensitive attribute (e.g., diagnosis). Table 2 shows the anonymized version of Table 1 with the value of *k* = 3.

The generated GQIs are distributed to the relevant data owners together with a list of data owners who have common GQIs. The list is then used by the data owners to connect with other data owners who have the same GQI. Every data owner then verifies their GQI with other data owners to form an equivalent group. Equivalent groups should satisfy the privacy policy of at least *k* data owner records present in every group. For example, Table 2 shows two equivalent groups that share the same GQI. The detailed steps of initialization for the data owner and data collector are shown in Algorithms 1, 2. Table 3 describes the symbols used in the algorithms.

**Algorithm 1:**
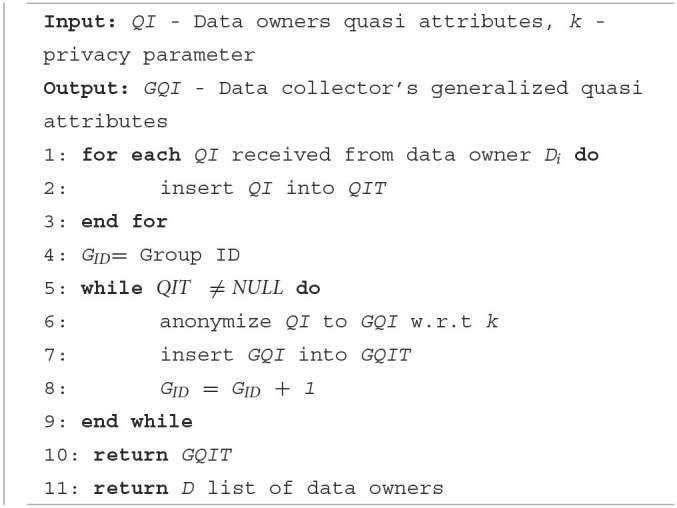
Data collector—initialization.

**Algorithm 2:**
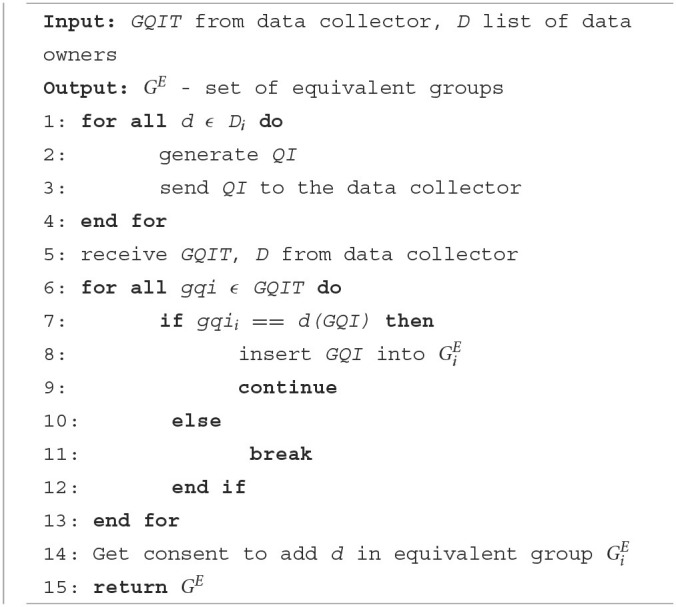
Data owner—initialization.

**Table 3:** Symbols.

**Symbols**	**Description**	**Symbols**	**Description**
*QI*	Quasi identifier	*R*_*GE*_	Number of records in *G*^*E*^
*QIT*	Quasi identifier table	*G*	Number of groups in anonymized dataset
*GQI*	Generalized quasi identifier	*L* ^1^	First leader
GQIT	Generalized quasi identifier table	*L* ^2^	Second leader
*G_*ID*_*	Group ID	*LT*	Leader information table
*D*	Data owner	*U* ^*ID*^	Group member user ID
*G* ^*E*^	Equivalent group	*CS*_*j*_	Counterfeit sensitive information of *L*^1^
*ST*_*R*_	Sensitive information of *L*^2^	*ST*_*j*_	Number sensitive information in *L*^1^
*AT*	Anonymized table	*S*_*j*_	Sensitive attribute in final table AT

Algorithm 1 runs at the data collector end to receive the quasi attributes from the data owners and to generate GQI based on any given anonymization techniques. It then disseminates the GQIs to the data owners. Algorithm 2 runs at the data owner's end to send the QIs to the data collector and to form equivalent groups based on the received GQI.

### 4.2. Leader election phase

On the data owners' side, equivalent classes are formed as per the privacy requirement *k*. In the leader election phase, two leaders are elected to represent the group and interact with the data collector. Algorithm 3 shows the detailed steps for leader election. First, the equivalent class members are counted. Then the *random()* function is used to generate two random numbers between 1 and the maximum number of members in the group. First, the randomly generated *userID* is considered as the first and second leader. Then we identified the energy and delay-less efficient leaders by utilizing the firefly-based algorithm proposed by Sarkar and Senthil Murugan (62). Firefly-based algorithm calculates the Euclidean distance between the elected leader and the nodes in the network then based on the distance metrics a firefly with cyclic randomization is performed to select the best leaders from among the groups. After every leader election, the leader table is updated. This algorithm ensures a single data owner is selected as the first and second leader. The elected leaders then transfer data to the data collector in the data collection phase.

**Algorithm 3:**
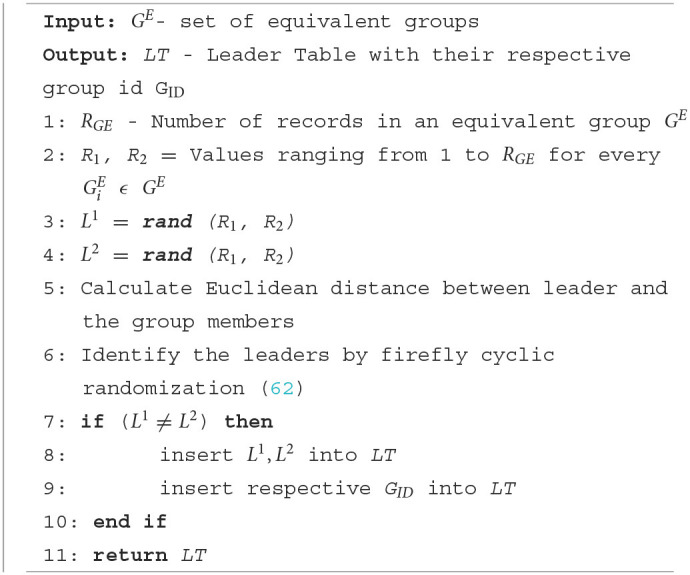
Leader election.

### 4.3. Data collection phase

The major task of the data collection phase is to collect the anonymized personal health records from the data owners. During the data collection initialization stage, QI attributes of data owners are generalized by the data collector then equivalent groups are formed on the data owners' side. To avoid explicit interaction of data owners with the data collector, group leaders are elected in the leader election phase. The leaders of each group are responsible for communicating QIs and sensitive identifiers. There are two leaders elected, the first leader (*L*^1^) is responsible to send the generalized QIs and multivalued sensitive attributes (MSA). The members equivalent group sends their anonymized records along with the multivalued sensitive attribute to the first leader. The MSA is a combination of an original sensitive attribute and *n*-1 counterfeit-sensitive attributes (where *n* is the size of the equivalent group's records). Hence, the first leader cannot discern the sensitive attributes of others in the group. Table 4 shows the example of the first leader anonymized dataset. The members of an equivalent class send their counterfeit sensitive attributes (CSA) (without the original sensitive attribute) to the second leader (*L*^2^). Table 5 shows the example of the second leader dataset that only contains CSA along with the userID.

**Table 4:** Anonymized data collection (first leader).

**User ID**	**Age**	**Sex**	**Weight**	**Diagnosis**
1,2,3	30–40	F	50–60	Gastritis, heart disease, pneumonia
		F		Flu, cancer, osteoarthritis
		F		Dyspepsia, gastritis, flu
4,5,6	55–65	M	65–75	Pneumonia, cancer, arrhythmia
		M		Flu, bronchitis, pneumonia
		M		Cancer, heart disease, gastritis

**Table 5:** Anonymized data collection (second leader).

**User ID**	**Diagnosis**
1,2,3	Heart disease, pneumonia
	Cancer, osteoarthritis
	Gastritis, flu
4,5,6	Cancer, arrhythmia
	Bronchitis, pneumonia
	Heart disease, gastritis

The data collector receives the datasets for the first and second leaders from each equivalent group during the data collecting phase. Elimination of counterfeit information from the first leader dataset is another important process for data collectors. It is hard for the data collector to identify the first and second leader datasets of each equivalent class as it performs subtraction and aggregation to eliminate the CSA from the dataset. The detailed steps of the data collection phase are given in Algorithm 4.

**Algorithm 4:**
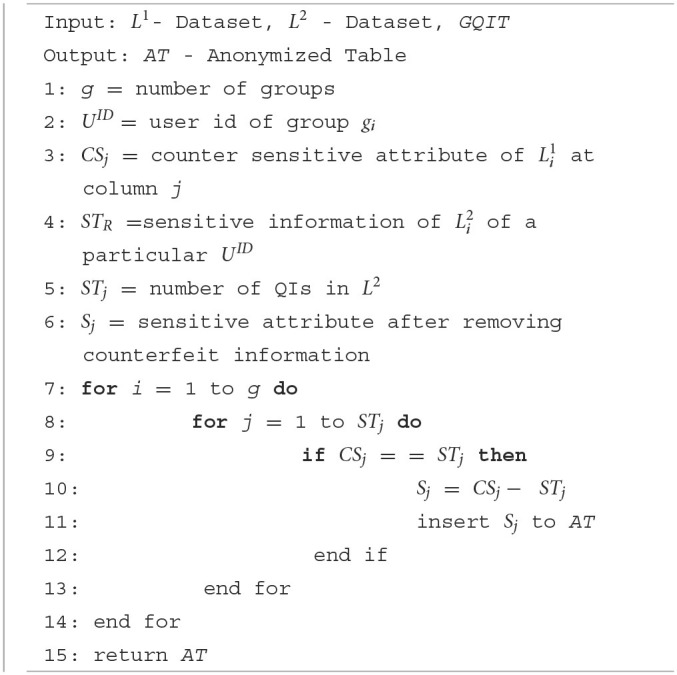
Data collection.

### 4.4. Dynamic data collection phase

The data collection protocol is designed in a way that it can consider data owners who join or depart the network dynamically. Dynamic data owners have to be efficiently managed to avoid any privacy breach to the network. The challenges with dynamic data owners are when a dynamic data owner joins the network, he/she should be placed in the appropriate equivalent group with minimal information loss and when a dynamic data owner leaves the network it should not affect the required privacy policy and without any privacy breach. During dynamic join or leave, the entire equivalent group needs to be reorganized, which incurs huge computational costs. Hence, the greedy heuristic method is proposed to efficiently handle dynamic data owners.

#### 4.4.1. Dynamic join

When dynamic data owners try to join the network, they transmit the data collector their QI attributes. The data collector considers the QI attribute as a dynamic join request and finds appropriate GQI from the existing GQIT to minimize the information loss. The new data owner is then added to the particular equivalent group who the share same GQI. The representatives (the first leader and the second leader) and group members are then notified about the new member in the group along with the modified GQI. Thereafter, the new data owner is considered for anonymization and GQI communication in the network.

#### 4.4.2. Dynamic leave

Data owners may leave the network due to unforeseen situations like power failure, system failure, and network failure. In such situations, a data owner leaves the network dynamically. It should be handled efficiently without breaching privacy. Each equivalent class consists of *k* or more data owners based on the privacy requirement. When a data owner departs the network, the corresponding equivalent class will be updated as per the number of remaining data owners to maintain the *k-*value for privacy. After the dynamic leave if the number of data owners is less than *k*, then the members of the equivalent group should be released to form a new group; otherwise, privacy would be breached. If a dynamic leave does not affect the minimum *k-*value of the group, then no specific handling is required as it is still within the privacy policy. But if the data owner who left is the first or second leader, then the leader election process should be carried out to elect new leaders.

Dismantling an existing equivalent group to form new groups during a dynamic leave is a heavy computational process. In the proposed protocol, such situations are handled by enforcing a threshold time limit. Dynamic leave of a data owner may be temporary or permanent. In temporary leave, the data owner rejoins the network within a particular time period, whereas, in permanent leave, the data owner will not join the network for further process. Hence, the threshold time is enforced to wait for any temporary leave data owner to rejoin. This reduces the computation cost as there is no further process required. If a data owner cannot rejoin within the time limit, then the members of the group will be released and a new group is formed based on the available data owners by satisfying the *k-*value and new leaders are elected. Thus, the dynamic leave of a data owner is efficiently handled in the protocol without a privacy breach.

## 5. Experiments

We evaluate our protocol in terms of computational complexity with respect to CSA elimination. In our privacy-preserving data collection protocol, we evaluate the computational complexity of the data collection phase only. The initialization and leader election phase has a complexity similar to traditional centralized anonymization techniques. Hence, the performance of the proposed protocol can be evaluated through CSA elimination of the data collection phase.

### 5.1. Experimental settings

The algorithms are implemented in Python programming and executed on Quad-Core Intel i7 at 2.2 GHz with 16 GB of RAM running Mac OS 10.15.3. We experimented our protocol on real-world public available datasets: the adult (63) and the informs (64).

### 5.2. Experimental analysis

**Figure 2:**
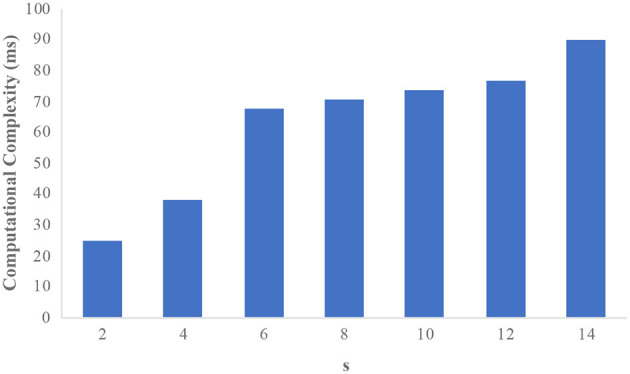
Computational complexity on the adult dataset.

The efficiency of the protocol in real-world datasets is analyzed in this section. First, the analysis is done with the adult dataset. There are 32,561 records with 14 attributes available in the adult dataset. The attributes “salary” and “occupation” are considered sensitive attributes. The sensitive attributes are merged as a single attribute “occupation-salary”; thus we increased the number of sensitive attributes to 30. It should be noted that our protocol does not consider multiple sensitive attributes. The computational complexity of the protocol is evaluated with the number of sensitive attributes (s) vs. time taken (in ms) by the protocol to eliminate the CSA. Figure 2 shows the computational complexity of the adult dataset with *s* as the *x*-axis and computational complexity (*ms*) as the *y*-axis. It is observed from the graph that the computational complexity increases with the number of sensitive attributes the protocol has to deal with is increased. Since the model deals with fewer sensitive attributes, the overhead seems to be stable with a slight increase in the *s* value.

**Figure 3:**
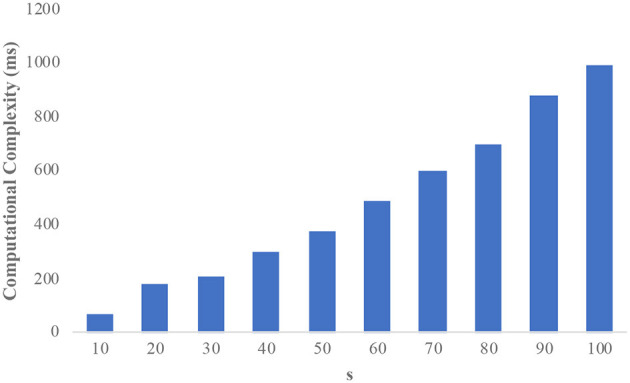
Computational complexity on the informs dataset.

**Figure 4:**
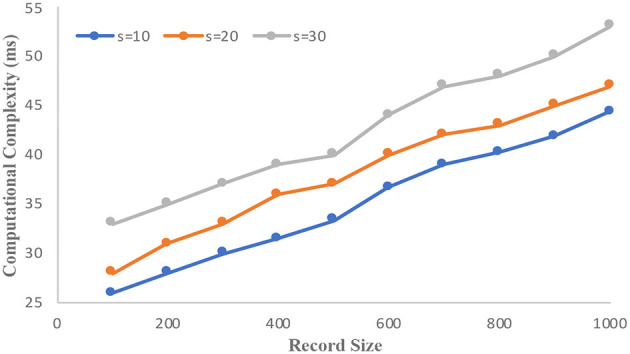
Computational complexity vs. record size.

The informs demographic dataset consists of 102,581 records and has 18 attributes. We consider “income” as the sensitive attribute and the domain size is 23,784. Figure 3 illustrates the computational complexity of the informs dataset. It is observed that the counterfeit elimination with larger domains incurs more overhead to the protocol. In the graph, the computational complexity constantly increases with the size of the sensitive attributes (s) in the network. Figure 4 illustrates how the informs dataset's computing complexity varies depending on the number of sensitive features. It is understood from the graphs that computational overhead increases with the size of the dataset and the domain size. The rise is caused by the volume of fake sensitive qualities that must be addressed.

### 5.3. Performance evaluation

**Figure 5:**
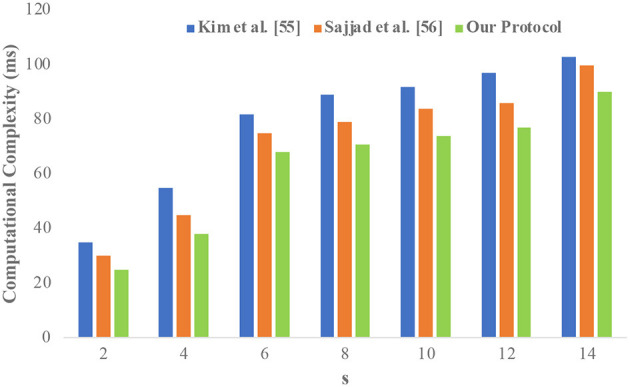
Performance evaluation of the proposed system.

The performance evaluation of the proposed study is compared with similar studies conducted by Kim and Chung (65) and Sajjad et al. (48). Figure 5 compares the performance of the proposed protocol with the state-of-the-art literature (experiments on the adult dataset). It is observed that the proposed protocol has considerably minimized the computational complexity. It is due to the slight changes in the CSA elimination where the distinct rows are compared instead of the whole dataset.

## 6. Discussion

This section outlines potential attacks on the suggested protocol as well as the measures the protocol uses to defend against them. We also discuss other important issues in the protocol such as leader collision mitigation and determination of CSA count. Furthermore, we discuss the complexity analysis and data utility of the protocol.

### 6.1. Internal and external identity disclosure attacks

When a legitimate member in the anonymization network tries to determine a person's identity, internal identity disclosure occurs. In our protocol, we consider a data collector as an adversary who seeks to gain more information about an individual. The adversary may target an individual to discern the sensitive attribute and to try to distinguish through the combination of quasi attributes. We employ a clustering-based *k*-anonymity (40) privacy model to anonymize personal health records that prevent identity disclosure. Clustering-based *k*-anonymity model generalizes the quasi attributes and forms clusters that contain at least *k* records each. As a result, the probability of identity disclosure is limited to *1/k* or less. Although the adversarial data collector has access to the generalized quasi attributes and sensitive attributes, the clustering-based *k*-anonymity policy makes internal identity disclosure nearly impossible.

External identity disclosure can happen when the data is transmitted using the given network. A practical data transmission environment is considered in the protocol, so it is necessary to add headers to the microdata. Our proposed protocol avoids direct connection between the data owner and collector in order to protect the external identity exposure, and instead relies on representatives (such as group leaders) to deliver the data to the data collector. Since all data owners in an equivalent group share the same generalized quasi attributes and the sensitive attributes are covered by a list of CSA, the group leaders are unable to determine who the data owners are. In addition to the original sensitive property, every record in the first leader dataset also contains at least *k-1* CSA. This ensures that the representative's identity disclosure does not exceed *1/k*.

### 6.2. Leader collision mitigation

Leader collision is a privacy attack where the elected representatives are adversarial data owners and attempt to discern sensitive information. In the leader election phase, each equivalent group elects two leaders. The first leader gathers the sensitive attribute along with the CSA. The second leader collects the CSA without real sensitive attributes and QIs. In an equivalent group if a single data owner is elected as the first and second leader, then the sensitive attributes can be discerned through the elimination of second leader sensitive attributes from the first leader dataset. In the proposed protocol, we verify the elected leaders' userIDs to make sure they are of a single data owner. Algorithm 3 shows the steps to elect different data owners as representatives.

Another type of LCA is identified by Sajjad et al. (48). Adversarial data owners may join the network by generating fake quasi attributes. They intend to be grouped under a particular equivalent group and try their chance to be elected as the group leaders. If both first and second leaders are elected from the adversarial data owners, they can collaborate and discern the sensitive attribute. This type of attack is called LCA. In our proposed protocol, we utilized firefly with a cyclic randomization algorithm (62) to elect the leaders. First, the number of data owners and their userIDs (index values) are collected, and based on the minimum and maximum index values, the random function generates two different userIDs. The generated userID is then considered the first and second leader for that specific data collection phase. The leader information is then stored in the leader information table for further verification.

### 6.3. Determination of counterfeit sensitive attribute count

Counterfeit sensitive attributes play an important role in protecting the sensitive attributes of the equivalent group. Similar privacy preserving data collection studies (48, 65) proposed the method of adding CSA to the anonymization network. However, the number of CSA to be added to the original sensitive attribute is not specified. It is important to determine the number of CSA required to protect the sensitive attribute in the anonymization network. In our protocol, we determine the count of CSA based on the privacy parameter *k*. It is proved from the *k*-anonymity-based privacy model that the identity can be disclosed only at the probability of 1/*k*. So, we consider the privacy parameter *k* as the count of CSA along with the actual sensitive attribute. Hence, the sensitive attribute of each data owner is protected and the probability to disclose the sensitive attribute is not >1/*k*. In our protocol, the privacy parameter value *k* is shared with every data owner as the CSA count. Each data owner generates *k*-1 counterfeit attributes to be added with the real sensitive attributes. To improve the quality of CSA, semantic diversity (66) among the sensitive attributes can be pitched in.

### 6.4. Complexity analysis

The complexity of the proposed protocol can be analyzed for the three phases of the data collection protocol: initialization, leader election, and data collection phase. The data owner's initialization phase comprises *QI* generation, submission, and *GQI* validation tasks. Let *Ct*_*gen*_, *Ct*_*sub*_, *and Ct*_*val*_ be the complexity of the three tasks. *QI* generation is the basic operation of the data owner, the cost *Ct*_*gen*_ is in *O*(1) where the *QI* is generated at a constant time. The complexity of *Ct*_*sub*_ is in *O*(1) where each data owner can submit the *QI* at a given time. *Ct*_*val*_ is in *O*(*k*) where *k* is the number of records in each equivalent group. In the data collector's initialization phase, the major tasks are *QI* generalization and *QI* distribution. Let *Ct*_*anon*_ and *Ct*_*dist*_ be the cost of the two tasks. *Ct*_*anon*_ is the cost of the anonymization technique that is adopted in the protocol. In traditional *k*-anonymity models, the cost of anonymization is NP-hard with complexity *O*(*n*^2^). In our proposed protocol, we adopted a clustering-based *k*-anonymity model so the cost *Ct*_*anon*_ is in $O\left(\frac{n^{2}}{k}\right)$. The distribution cost *Ct*_*dist*_ is in *O*(*n*) where *n* is the number of data owners in the network. The total cost of the data collector at the initialization stage is in $O\left(\frac{n^{2}}{k}\right)+O\left(n\right)$. Leader election is another trivial task, the cost of *Ct*_*elec*_ is in *O*(*u*), where *u* denotes the users in the network. In an equivalent group, *Ct*_*elec*_ is in *O*(*k*), where *k* is the records in the equivalent class.

In the data collection phase, the elimination of CSA from the first leader dataset using the dataset of the second leader is a major task. The CSA values obtained from the second leader dataset are required to be compared with anonymized records of the first leader dataset. Let *s* be the sensitive attributes in an equivalent group then the number of sensitive attributes in a group is *k*×*s*. The list of CSA in the dataset is *k*×*s*− 1. If *g* is the number of equivalent classes, then the cost of CSA elimination is *O*(*g* · *k*^2^ · *s*^2^). In our protocol, counterfeit elimination is carried out by comparing the CSA only with distinct sensitive attributes. Hence, the cost of CSA elimination is restricted to *O*(*kds*) where *d* denotes the sensitive attribute domain size.

### 6.5. Data utility

In the process of anonymization, the original dataset tends to suffer from poor data utility. The data utility is generally measured through various information loss metrics. Likewise, a dataset with minimum or no information loss may leak privacy. Hence, it is important to maintain the trade-off between privacy and data utility. In our protocol, the anonymization process is carried out only during the initialization phase. The QI attributes are anonymized by the data collector through a utilized clustering-based *k*-anonymity model (53) that forms clusters as the equivalent groups with *k* or more records in each group. Thus, data utility is inherited from the adopted privacy model. Furthermore, our protocol can adopt any *k*-anonymity based privacy model. The information loss and data utility are based on the chosen privacy model. Hence, in this study, we did not present the results of the information loss as our protocol is independent of the privacy model.

### 6.6. Healthcare data security analysis

Beyond privacy protection, it is also essential to secure healthcare data from unauthorized access and disclosure (67). The potential security threats to a healthcare system are covered in this section.

Due to the requirements of the legal, ethical, and medical domains, healthcare data must be protected from unauthorized access and disclosure (68). To protect health information, three data security techniques are widely in use; they are cryptographic security, blockchain based security, and network security. Cryptography is the most commonly used technique to protect data from unauthorized access, tampering, and an interception. Data encryption plays a major role in protecting data. Qiu et al. (69) proposed a selective encryption algorithm to secure healthcare data sharing with fragmentation and dispersion techniques. This algorithm ensures data safety even when the cloud servers and keys are compromised. Blockchain based security techniques are popular because of their unhackable distributed ledger and smart contracts. Zhuang et al. (70) proposed a blockchain model to protect patient records from unauthorized access and disclosure. The blockchain properties such as immutability, smart contract, and distributed ledgers ensure data consistency, quick access, and patient authorization. The network is another essential part of the healthcare domain that needs proper security to avoid eavesdropping, intrusion, and tampering attacks. Most healthcare systems employ IoT, wireless networks, and body area networks. So appropriate network security is required to protect the data transferred between the data owner and the collector (71–73).

## 7. Conclusion and future work

In this article, we presented a privacy-preserving healthcare data collection protocol. The state-of-the-art privacy-preserving data collection models, coerce strict assumptions such as secure private channels or third-party anonymization between the data owners and the collector. The proposed protocol eliminates such assumptions and offers anonymous data collection through the elected representatives among the data owners. The protocol is efficient in tackling internal and external identity disclosure through an adopted clustering-based *k*-anonymity model. We proposed solutions for possible collisions among the elected representatives within the equivalent group. We also proposed a new efficient method to add CSA to protect the real sensitive attributes. Furthermore, dynamic data owners are efficiently handled in the protocol by a greedy heuristic method. Through extensive experimental analysis, we proved that our protocol incurs considerably minimum computational complexity compared with state-of-the-art techniques. This makes our protocol more suitable for collecting huge amounts of healthcare datasets without privacy breach. Our protocol is built to accommodate any *k*-anonymity-based privacy models; hence, the data utility can be optimized as per the requirement.

We intend to conduct several future studies to address the limitations of this study. First, we would like to focus on minimizing the other privacy risks such as attribute disclosure, membership disclosure, and similarity attacks. Currently, our study is focused mainly on protecting personal data from identity disclosure. Considering other privacy attacks would make our protocol more robust for healthcare data collection. Second, we would like to employ anonymization techniques other than *k*-anonymity such as bucketization and anatomy to enhance the data utility of the protocol.

## Data availability statement

Publicly available datasets were analyzed in this study. This data can be found here: https://archive.ics.uci.edu/ml/datasets/adult.

## Author contributions

JA and JK conceived the idea and worked on the technical details. JA, RE, and JK devised the work, the main conceptual ideas, the proof outline, and worked on the manuscript. All authors contributed to the article and approved the submitted version.
